# Distinct systemic cytokine networks in symptomatic and asymptomatic carotid stenosis

**DOI:** 10.1038/s41598-020-78941-8

**Published:** 2020-12-15

**Authors:** Ricarda D. Stauss, Gerrit M. Grosse, Lavinia Neubert, Christine S. Falk, Danny Jonigk, Mark P. Kühnel, Maria M. Gabriel, Ramona Schuppner, Ralf Lichtinghagen, Mathias Wilhelmi, Karin Weissenborn, Claudia Schrimpf

**Affiliations:** 1 Department of Neurology, Hannover Medical School, Carl-Neuberg-Str. 1, 30625 Hannover, Germany; 2 Institute of Pathology, Hannover Medical School, Hannover, Germany; 3 Institute of Transplant Immunology, Hannover Medical School, Hannover, Germany; 4 Institute of Clinical Chemistry, Hannover Medical School, Hannover, Germany; 5 Department of Vascular- and Endovascular Surgery, St. Bernward Hospital, Hildesheim, Germany; 6 Division of Vascular and Endovascular Surgery, Department of Cardiothoracic-, Transplantation- and Vascular Surgery, Hannover Medical School, Hannover, Germany; 7 Department of Vascular Surgery, University Hospital Zurich, Zurich, Switzerland

**Keywords:** Cerebrovascular disorders, Neurovascular disorders, Stroke, Biomarkers, Atherosclerosis, Carotid artery disease, Embolism, Thromboembolism, Cytokines

## Abstract

Inflammatory processes are crucial in atherosclerosis and atherothrombosis. This study aimed to identify a cytokine-pattern that is associated with plaque-vulnerability or symptomatic state in comprehensively investigated patients with symptomatic (sCS) and asymptomatic carotid stenosis (aCS). Twenty-two patients with sCS and twenty-four patients with aCS undergoing carotid endarterectomy (CEA) were considered. A cytokine-panel was measured in plasma-specimens prior to surgery and at a 90 day follow-up. Doppler-ultrasound detecting microembolic signals (MES) in the ipsilateral middle cerebral artery was performed. Carotid plaques were analysed regarding histopathological criteria of plaque-vulnerability and presence of chemokine receptor CXCR4. Correction for multiple comparisons and logistic regression analysis adjusting for vascular risk factors, grade of stenosis, antithrombotic and statin pretreatment were applied. In sCS-patients higher plasma-levels of Fractalkine (CX_3_CL1), IFN-α2, IL-1β, IL-2, IL-3, IL-7 were found compared to aCS-patients. CXCR4-expression on inflammatory cells was more evident in sCS- compared to aCS-plaques and was associated with vulnerability-criteria. In contrast, plasma-cytokine-levels were not related to CXCR4-expression or other vulnerability-criteria or MES. However, in both groups distinct inter-cytokine correlation patterns, which persisted at follow-up and were more pronounced in the sCS-group could be detected. In conclusion, we identified a distinct cytokine/chemokine-network in sCS-patients with elevated and closely correlated mediators of diverse functions.

## Introduction

Approximately 10–15% of ischaemic strokes and transient ischaemic attacks (TIA) are attributable to thromboembolism due to ipsilateral carotid artery stenosis^1^. Carotid endarterectomy (CEA) or Carotid Artery Stenting (CAS) are recommended for secondary stroke prevention in patients with symptomatic carotid stenosis (sCS)^2^. In patients with asymptomatic carotid stenosis (aCS) indication for prophylactic CEA is controversial, since stroke rates are similar compared to best medical treatment^3^. Therefore, the identification of patients with aCS who are at a higher risk for stroke is crucial in order to select a subgroup that benefits from primary preventive surgery.

Broad evidence exists that in-plaque inflammation is associated with a higher risk of plaque destabilisation, rupture and subsequent risk for thromboembolism—a state often referred to as “vulnerable”^4^. Circulating cytokines are important mediators of inflammatory cell influx into the vessel wall and thus crucial in atherosclerotic disease burden^5^. When plaque rupture occurs, platelets attach to sub-endothelial matrix molecules at the lesion side, followed by further coagulation pathways^6^. Again, inflammatory cytokines play an important role in this atherothrombotic process^6,7^. In particular, inflammation serves as a trigger for coagulation, while activated platelets also secrete cytokines in terms of a self-reinforcing process^8^. Deeper knowledge of the thrombo-inflammatory processes associated with cerebrovascular events in patients with carotid artery stenosis might lead to novel diagnostic as well as therapeutic strategies^8^. Previous studies rather focused on the identification of novel biomarkers of plaque vulnerability in previously asymptomatic stenoses and less on possible associations with the occurrence of actual neurological symptoms due to CS. In this study, we therefore aimed to investigate patients with aCS and sCS in order to identify a cytokine pattern that is associated with symptomatic state or with characteristics of plaque vulnerability in order to establish a biomarker-based approach for risk stratification in patients with CS.

## Methods

### Study population

Between July 2017 and June 2018 50 patients with aCS or sCS planned for CEA were prospectively recruited at the Department of Neurology and the Department of Cardiothoracic-, Transplantation- and Vascular Surgery at Hannover Medical School. A carotid stenosis was defined as “symptomatic” after exclusion of competing stroke etiologies if an ischaemic stroke, a transient ischaemic attack (TIA), amaurosis fugax or central retinal artery occlusion occurred in the territory of the affected artery. Stroke diagnostics including cranial computed tomography (CCT) and/or magnetic resonance imaging (MRI), CT or MR angiography, transthoracic or transesophageal echocardiography, cardiac rhythm monitoring for at least 24 h and Doppler/duplex ultrasound were performed in all patients with sCS. Further exclusion criteria in the whole collective were acute infections, immunological disease, immunosuppressive therapy or current malignant disease. The study was approved by the ethics committee at Hannover Medical School (Ethics vote no. 7484-2017) and performed according to the ethical principles of the Declaration of Helsinki. All patients gave written informed consent before being included in the study.

### Clinical data

Patients’ demographical and clinical data were collected following a standardised interview including cardiovascular risk factors that were subsumed in the Essen Stroke Risk Score (ESRS), degree of carotid artery stenosis and current medication. On admission, stroke severity was quantified by the National Institutes of Health Stroke Scale (NIHSS) in sCS patients. Carotid artery stenosis was detected in cervical MR- or CT-angiography as well as duplex ultrasound and classified according to the North American Symptomatic Carotid Endarterectomy Trial (NASCET) criteria^9^. Degree of stenosis was defined as moderate (50–69%), severe (70–90%) or highly severe (> 90%). The clinical outcome was assessed three months after the date of surgery in a follow-up examination using the NIHSS and modified Rankin Scale (mRS).

### Transcranial Doppler monitoring

Thirty-four patients with sufficient transtemporal window underwent a standardised preoperative transcranial Doppler monitoring of the ipsilateral middle cerebral artery (MCA) according to the criteria of the Consensus on Microembolus Detection by TCD^10^. The MCA was insonated via the transtemporal window in 45 to 55 mm depth using a 2-MHz transducer. Monitoring was recorded up to 1 h unless it had to be stopped earlier due to patients’ incompliance. Microembolic signals (MES) were identified visually and audibly by an investigator blinded to clinical data.

### Biomarkers

Peripheral venous blood was drawn prior to surgery to minimize the influence of possible post-ischaemic neuroinflammation. Additionally, blood was drawn at a 3 months follow-up. Specimens were centrifuged at 3000*g* for 15 min and plasma stored at − 80 °C for further analysis. Cytokines and chemokines were quantified using Luminex-based MILLIPLEX MAP Human Cytokine and Human sepsis Panels (Merck Millipore, Darmstadt, Germany) in EDTA-Plasma according to the manufacturer’s instructions. The following biomarkers were considered for analysis: Epidermal growth factor (EGF), Fibroblast growth factor 2 (FGF-2), Eotaxin (CCL11), Transforming growth factor alpha (TGF-α), Granulocyte colony stimulating factor (G-CSF), Granulocyte macrophage colony stimulating factor (GM-CSF), Fractalkine (CX_3_CL1), Interferon alpha 2 (IFN-α2), Interferon gamma (IFN-γ), Growth regulated oncogene (GRO-α, CXCL1), Interferon gamma induced protein 10 (IP-10, CXCL10), Macrophage derived chemokine (MDC, CCL22), Interleukin-12p70 (IL-12p70), Soluble CD40-ligand (sCD40L), Interleukin-17A (IL-17A), Interleukin-1 receptor antagonist (IL-1RA), Interleukin-1 alpha (IL-1α), Interleukin-1 beta (IL-1β), Interleukin-2 (IL-2), Interleukin-3 (IL-3), Interleukin-4 (IL-4), Interleukin-5 (IL-5), Interleukin-6 (IL-6), Interleukin-7 (IL-7), Interleukin-8 (IL-8, CXCL8), Interleukin-9 (IL-9), Interleukin-10 (IL-10), Interleukin-13 (IL-13), Interleukin-15 (IL-15), Monocyte chemotactic protein 1 (MCP-1, CCL2), Monocyte chemotactic protein 3 (MCP-3, CCL7), Macrophage inflammatory protein 1 alpha (MIP-1α, CCL3), Macrophage inflammatory protein 1 beta (MIP-1β, CCL4), Tumor necrosis factor alpha (TNF-α), FMS-like tyrosine kinase-3 ligand (Flt-3L), Interleukin-12p40 (IL-12p40), Macrophage inhibitory factor (MIF), Tissue plasminogen activator inhibitor (tPAI), Soluble intercellular adhesion molecule-1 (sICAM-1), Soluble vascular adhesion molecule 1 (sVCAM-1). Standard curves and concentrations were calculated with Bio-Plex-Manager 6.2 software (Bio-Rad Laboratories, Hercules, CA, USA).

### Histological analysis

**Figure 1:**
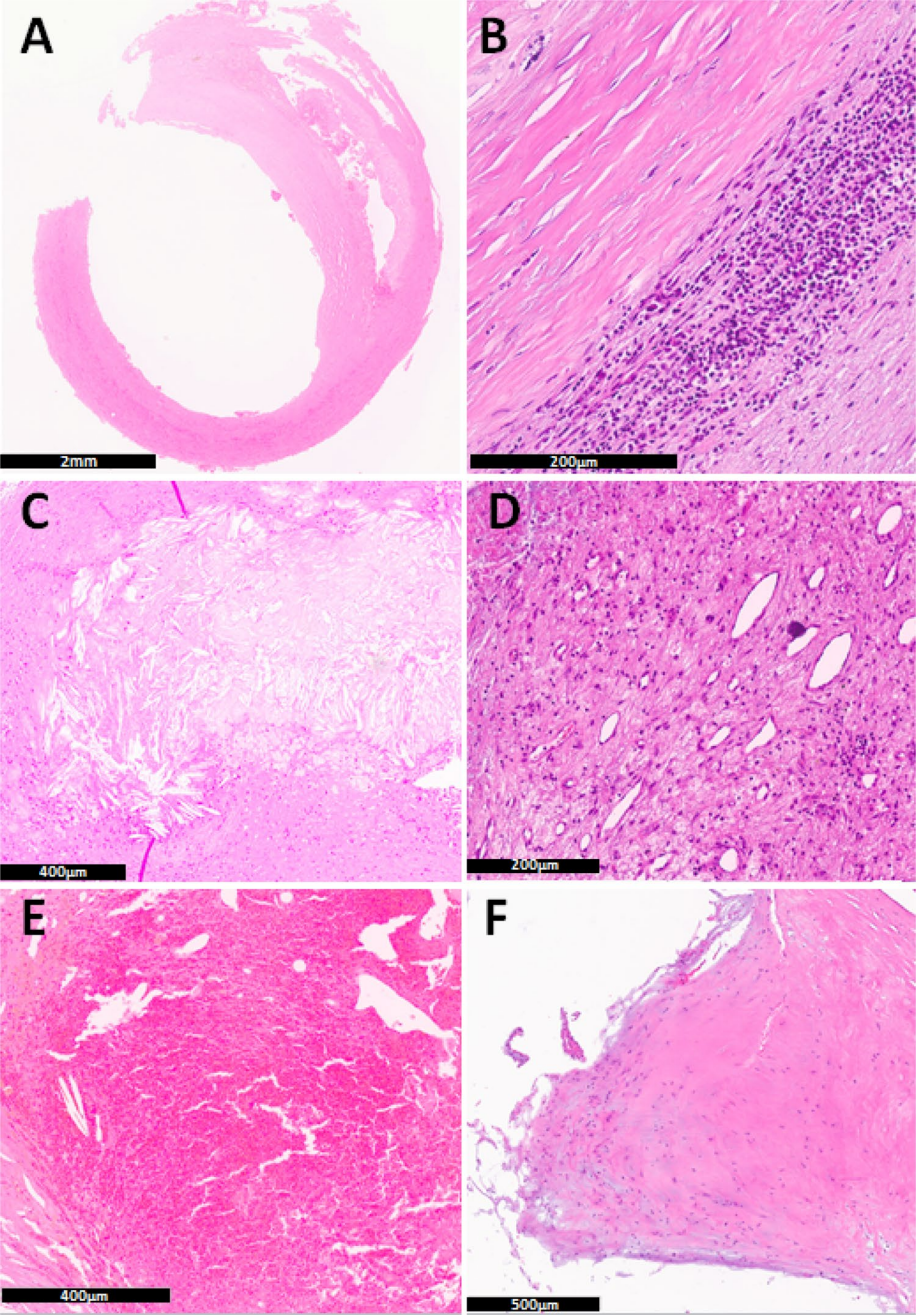
Histological criteria of plaque vulnerability. Representative sections displaying criteria of the histological sum score. Carotid plaque, HE-staining (overview, scale bar: 2 mm) (**A**). Representative areas of different specimens showing chronic inflammation (scale bar: 200 µm) (**B**), cholesterol crystals (scale bar: 400 µm) (**C**), neovascularization (scale bar: 200 µm) (**D**) intraplaque hemorrhage (scale bar: 400 µm) (**E**) and an adherent thrombus (scale bar: 500 µm) (**F**).

Carotid plaques from all patients were obtained after CEA and analysed at the Institute of Pathology at Hannover Medical School by an observer blinded to clinical characteristics. Specimens were fixed in 4% buffered formalin immediately after removal, then embedded in paraffin. 2 µm thick sections were cut from the tissue sections followed by histological staining using Hematoxylin and Eosin, Elastica van Gieson (EvG) and Periodic Acid Schiff (PAS). Immunohistochemistry was performed according to established protocols using anti-CXCR4 antibody (Abcam, Cambridge, MA, USA). A representative section showing maximal atherosclerotic alteration was analysed regarding the following histological criteria: intima fibrosis, lipid core, calcification, media degradation, acute and chronic inflammation, cholesterol crystals, neovascularization, intraplaque hemorrhage, fibrous cap rupture and adherent thrombus. For immunohistochemical analysis the number of CXCR4-positive macrophages in a 400 × high-power field (HPF) was counted, representing the area of maximum positive staining in each section. Furthermore, the presence of CXCR4-positivity in inflammatory cells was assessed as a dichotomized parameter. The count of neovessels was quantified as the median of ten 10 × HPFs. We established a semiquantitative histological score containing the following items of advanced criteria of plaque-vulnerability: chronic inflammation, cholesterol crystals, neovascularization, intraplaque hemorrhage, adherent thrombus (Fig. 1). The median sum score was used as a cut off-value to stratify the samples into two groups, according to their individual sum of vulnerability criteria (< 2 points vs. ≥ 2 points).

### Statistical analysis

Normal distribution was tested using the Kolmogorov–Smirnov test. Group differences were analysed with Student’s *t* test for normally distributed or Mann–Whitney *U* test for non-normally distributed data. For group comparisons the Benjamini–Hochberg procedure was applied using False Discovery Rate (FDR) estimation in order to correct for multiple comparisons. Chi-square test was used to compare categorical data. Binary logistic regression was done using the stepwise backwards method and included grade of stenosis, ESRS, antithrombotic pretreatment and statin pretreatment (i.e. treatment that was initiated before the event/hospitalization) as those parameters were significantly different at baseline. Correlations were calculated with Spearman rho test. Receiver Operating Characteristic (ROC) analysis was conducted to investigate the discriminative value of cytokines between groups. Statistical analysis was performed using IBM SPSS Statistics 26 (SPSS Inc. Chicago, IL, USA). Figures were created using GraphPad Prism 5 (GraphPad Inc., La Jolla, CA, USA) and Gephi.

## Results

### Patients’ characteristics

Of 50 patients recruited, 4 patients had to be excluded due to the following reasons: One patient withdrew consent, one patient was diagnosed with malignant disease and one presented a competing stroke etiology in stroke diagnosis. In another case the carotid plaque specimen was not collected leading to case exclusion. Thus, 22 patients with sCS and 24 patients with aCS were available for analysis. For demographic and clinical data see Table 1. Significant group differences existed regarding the following parameters: coronary artery disease (p = 0.006), previous myocardial infarction (p = 0.028), degree of stenosis (p = 0.023), ESRS (p = 0.002), antithrombotic pretreatment (p < 0.001) and statin pretreatment (p = 0.008), with more severe stenosis, increased morbidity and intensified medication in asymptomatic patients. In the sCS group 7 patients had transient symptoms, 4 patients suffered central retinal artery occlusion. Likewise, stroke severity was low with a median NIHSS of 0. The median time difference between ischaemic event and preoperative blood collection was 8 days in the sCS group. Three patients declined the follow-up examination. The median time to follow-up was 95 days and did not significantly differ between the groups (p = 0.234).

**Table 1:** Patients’ demographic and clinical characteristics.

	sCS	aCS	p-value
	n = 22	n = 24	
Age (years), mean ± SD	66.27 ± 9.44	70.54 ± 9.38	0.131
Sex			0.066
Male (%)	19 (86.4)	15 (62.5)	
Female (%)	3 (13.6)	9 (37.5)	
BMI (kg/m^2^) ± SD	27.34 ± 3.37	29.38 ± 5.74	0.155
Adiposity (%)	3 (13.6)	9 (37.5)	0.066
ESRS on admission (Q1-Q3)	3 (2–4)	4 (4–5)	0.002
ESRS at 90 days (Q1-Q3)	4 (3–4)	4 (4–5)	0.083
Hypertension (%)	16 (72.7)	21 (87.5)	0.207
Diabetes mellitus (%)	5 (22.7)	8 (33.3)	0.425
Dyslipoproteinemia (%)	18 (81.8)	19 (79.2)	0.821
Peripheral arterial disease (%)	2 (9.1)	7 (29.2)	0.086
Nicotine abuse (%)	17 (77.3)	19 (79.2)	0.881
Alcohol abuse (%)	4 (18.2)	5 (20.8)	0.821
Coronary artery disease (%)	2 (9.1)	11 (45.8)	0.006
Previous myocardial infarction (%)	1 (4.5)	7 (29.2)	0.028
Atrial fibrillation (%)	0 (0)	2 (8.3)	0.166
Previous stroke (%)	3 (13.6)	5 (20.8)	0.520
Dementia (%)	0 (0)	1 (4.2)	0.333
Epilepsy (%)	2 (9.1)	1 (4.2)	0.499
Medication			
Antithrombotic pretreatment (%)	9 (40.9)	23 (95.8)	< 0.001
Statin pretreatment (%)	8 (36.4)	18 (75.0)	0.008
NIHSS on admission (Q1–Q3)	0 (0–2)	N.A	
NIHSS 90 days (Q1–Q3)	0 (0–1)	N.A	
mRS 90 days (Q1–Q3)	0 (0–1.25)	N.A	
Grade of stenosis			0.023
Moderate	1 (4.5)	0 (0)	
Severe	10 (45.5)	20 (83.3)	
Highly severe	11 (50.0)	4 (16.7)	
Contralateral CS	13 (59.1)	8 (33.3)	0.08
Contralateral CO	1 (4.5)	3 (12.5)	0.339
Time from ischaemic event to blood collection (days) (Q1–Q3)	8 (3.5–12)	N.A	
Time to follow-up (days) (Q1–Q3)	92 (90–104)	97 (91–104)	0.234

P-values were calculated using Student’s *t* test, Mann–Whitney *U* test or Chi-square test, as appropriate. *aCS* asymptomatic carotid artery stenosis, *BMI* body mass index, *ESRS* Essen stroke risk score, *CO* carotid occlusion, *CS* carotid stenosis, *NIHSS* National Institutes of Health Stroke Scale, *mRS* modified Rankin Scale, *Q1–Q3* quartile 1–quartile 3, *sCS* symptomatic carotid artery stenosis, *SD* standard deviation.

### Microembolic signals

TCD monitoring was performed in 34 patients. Reasons for missing TCD-monitoring were missing written consent for this examination (n = 2), absent temporal window (n = 5), emergency operation (n = 2) and incompliance leading to discontinuation (n = 3). Symptomatic patients showed a range of 0–13 MES/h with a median count of 0 MES/h, asymptomatic patients showed a range of 0–2 MES/h with a median count of 0 MES/h (p = 0.707). The count of MES per hour did not correlate with plasma cytokine concentrations. In addition, we divided the study cohort into two groups: Patients with MES at baseline (MES+) and patients without MES (MES−). We did not find group differences regarding cytokine levels or histological features.

### Preoperative biomarker levels

**Figure 2:**
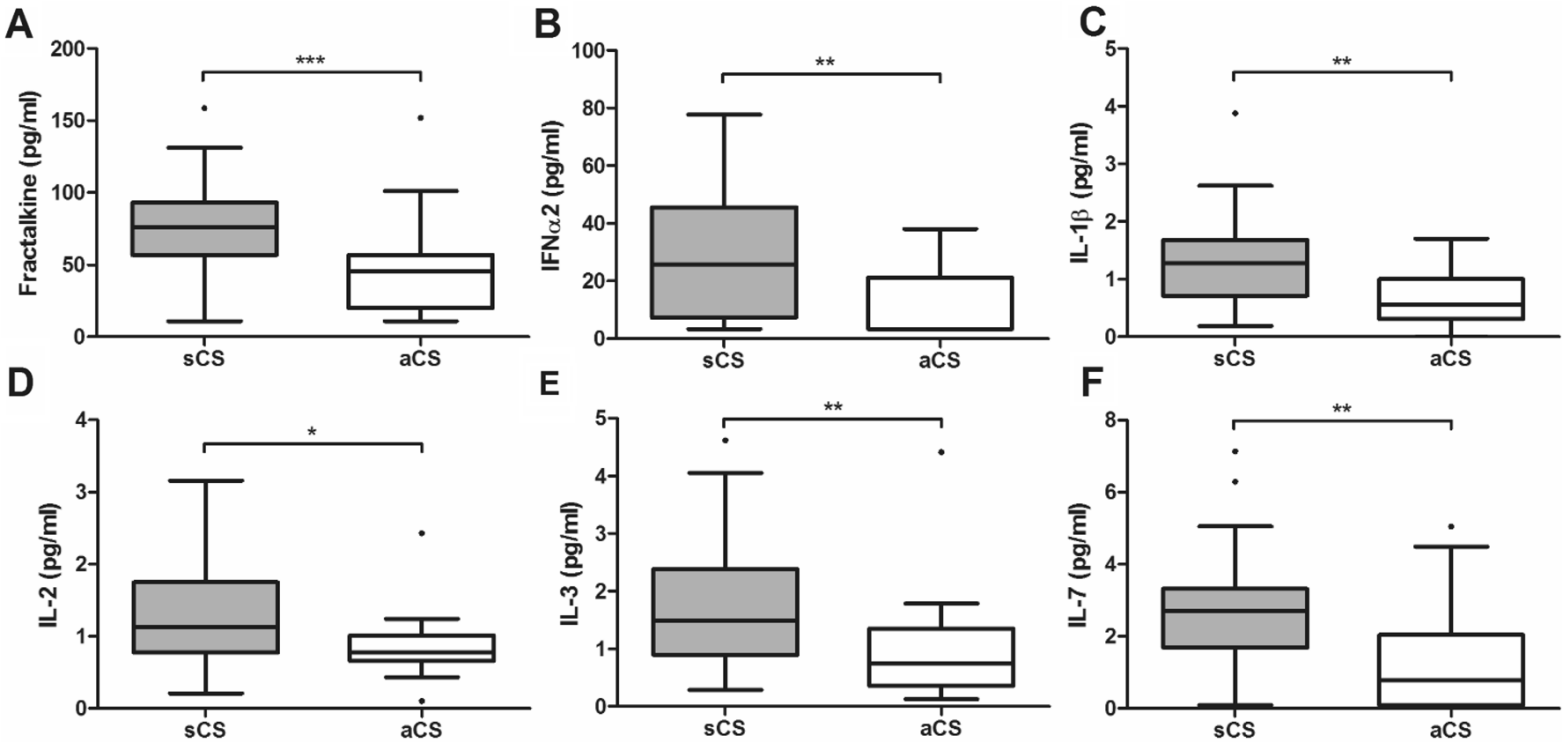
Differences of cytokine concentrations between patients with symptomatic and asymptomatic carotid stenosis. Boxplots depicting a comparison of plasma cytokine levels in patients with symptomatic (sCS) and asymptomatic carotid stenosis (aCS). After correction for multiple comparisons and adjustment for possible confounders significant differences were observed concerning Fractalkine (**A**), IFN-α2 (**B**), IL-1β (**C**), IL-2 (**D**), IL-3 (**E**), IL-7 (**F**). Asterisks refer to univariable analysis (***p < 0.001; **p < 0.01; *p < 0.05).

At baseline, in total 44 samples were available. For the follow-up 42 samples could be analysed. Due to many values below minimum detection levels following analytes needed to be excluded from further analysis: Flt3L, MCP-3, IL-12p40, IL13, IL-1α, IL-9, IL-6, TNF-b, VEGF, leaving 33 analytes for further analysis. Results of biomarker analysis are listed in Table 2. In univariable analysis of preoperative samples sCS-patients had significantly higher plasma concentrations of the following cytokines than aCS-patients regarding preoperative blood samples: EGF (p = 0.029), FGF2 (p = 0.032), TGF-α (p = 0.034), GM-CSF (p = 0.023), Fractalkine (CX_3_CL1) (p < 0.001), IFN-α2 (p = 0.003), IFN-γ (p = 0.038), IL-1β (p = 0.007), IL-2 (0.010), IL-3 (p = 0.006), IL-5 (p = 0.040), IL-7 (p = 0.002), IL-8/CXCL8 (p = 0.009), IL-10 (p = 0.037), IL-15 (p = 0.018), IL-17A (p = 0.019), MIP-1α/CCL3(p = 0.020). Additionally, a non-significant difference by trend existed for GRO-α/CXCL1 (p = 0.082) and G-CSF (p = 0.081). After applying Benjamini–Hochberg procedure and binary logistic regression analysis adjusted for degree of stenosis, ESRS, antithrombotic pretreatment and statin pretreatment following markers remained significantly different: Fractalkine (p = 0.036), IFN-α2 (p = 0.023), IL-1β (p = 0.025), IL-2 (p = 0.023), IL-3 (p = 0.030) and IL-7 (p = 0.029) (Fig. 2). ROC-analysis revealed following areas under the curve (AUC): Fractalkine/CX_3_CL1: 0.806, IFN-α2: 0.758, IL-1β: 0.738, IL-2: 0.725, IL-3: 0.743, IL-7: 0.769.

**Table 2:** Comparison of biomarker concentrations in patients with symptomatic and asymptomatic carotid stenosis.

	sCS	aCS	p-value univariate analysis	p-value logistic regression analysis
**EGF (pg/ml)**				
Preoperative sample [median (Q1–Q3)]	12.44 (03.08–26.12)	1.25 (1.25–16.1)	0.029	0.048
90 days follow-up sample [median (Q1–Q3)]	21.78 (11.42–62.14)	9.76 (2.81–20.77)	0.010	0.114
**FGF-2 (pg/ml)**				
preoperative sample [median (Q1–Q3)]	46.28 (30.13–63.88)	31.25 (21.37–49.51)	0.032	0.016
90 days follow-up sample [median (Q1–Q3)]	45.44 (30.10–61.89)	28.94 (21.37–54.06)	0.134	0.150
**Eotaxin (pg/ml)**				
preoperative sample [median (Q1–Q3)]	67.38 (41.91–85.13)	48.59 (35.24–88.56)	0.806	0.124
90 days follow-up sample [median (Q1–Q3)]	69.09 (45.38–94.37)	61.88 (42.36–87.15)	0.809	0.282
**TGF-α (pg/ml)**				
preoperative sample [median (Q1–Q3)]	0.56 (0.41–0.79)	0.30 (0.22–0.70)	0.034	0.669
90 days follow-up sample [median (Q1–Q3)]	0.54 (0.34–0.67)	0.32 (0.15–0.55)	0.075	0.697
**G-CSF (pg/ml)**				
preoperative sample [median (Q1–Q3)]	11.45 (2.38–26.52)	2.38 (2.38–22.75)	0.081	0.727
90 days follow-up sample [median (Q1–Q3)]	22.75 (11.99–37.92)	10.65 (4.53–29.68)	0.080	0.225
**GM-CSF (pg/ml)**				
preoperative sample [median (Q1–Q3)]	4.71 (1.48–7.67)	2.11 (0.22–4.05)	0.023	0.025
90 days follow-up sample [median (Q1–Q3)]	5.04 (1.55–9.34)	2.43 (1.47–5.28)	0.090	0.082
**Fractalkine (pg/ml)**				
preoperative sample [median (Q1–Q3)]	75.89 (56.70–93.17)	45.99 (19.91–56.70)	< 0.001	0.036
90 days follow-up sample [median (Q1–Q3)]	71.25 (56.70–101.30)	64.17 (34.07–93.17)	0.071	0.245
**IFN-α2 (pg/ml)**				
preoperative sample [median (Q1–Q3)]	25.81 (7.33–45.50)	3.36 (3.36–21.23)	0.003	0.023
90 days follow-up sample [median (Q1–Q3)]	23.52 (8.79–44.19)	10.60 (3.36–25.81)	0.043	0.056
**IFN-γ (pg/ml)**				
preoperative sample [median (Q1–Q3)]	8.95 (2.61–11.98)	3.71 (0.78–7.07)	0.038	0.632
90 days follow-up sample [median (Q1–Q3)]	7.26 (1.97–9.52)	4.09 (1.51–8.95)	0.421	
**GRO-α (pg/ml)**				
preoperative sample [median (Q1–Q3)]	695.62 (416.49–1138.17)	476.72 (239.53–875.61)	0.082	0.393
90 days follow-up sample [median (Q1–Q3)]	610.18 (368.58–1319.76)	965.39 (513.93–1337.75)	0.388	0.451
**IP-10 (pg/ml)**				
preoperative sample [median (Q1–Q3)]	330.00 (286.24–599.50)	397.59 (253.04–464.90)	0.378	0.735
90 days follow-up sample [median (Q1–Q3)]	288.95 (216.34–642.74)	441.40 (308.93–671.78)	0.155	0.860
**MDC (pg/ml)**				
preoperative sample [median (Q1–Q3)]	420.80 (318.65–473.98)	425.40 (313.56–564.65)	0.838	0.176
90 days follow-up sample [median (Q1–Q3)]	466.21 (341.28–556.46)	449.37 (265.08–611.50)	0.739	0.532
**IL-12p70 (pg/ml)**				
preoperative sample [median (Q1–Q3)]	0.09 (0.09–0.98)	0.09 (0.09–0.67)	0.412	0.985
90 days follow-up sample [median (Q1–Q3)]	0.09 (0.09–0.86)	0.09 (0.09–0.59)	0.560	0.385
**sCD40L (pg/ml)**				
preoperative sample [median (Q1–Q3)]	123.98 (73.63–271.08)	94.96 (70.94–286.46)	0.622	0.646
90 days follow-up sample [median (Q1–Q3)]	165.71 (86.35–363.85)	120.04 (42.05–201.68)	0.091	0.212
**IL-17A (pg/ml)**				
preoperative sample [median (Q1–Q3)]	2.31 (1.37–2.58)	1.33 (0.89–2.06)	0.019	0.763
90 days follow-up sample [median (Q1–Q3)]	2.21 (0.72–3.10)	1.18 (0.75–2.32)	0.262	0.745
**IL-1RA (pg/ml)**				
preoperative sample [median (Q1–Q3)]	5.22 (1.15–17.77)	2.44 (0.02–7.79)	0.142	0.103
90 days follow-up sample [median (Q1–Q3)]	5.95 (0.02–13.49)	3.13 (0.02–7.80)	0.445	0.087
**IL-1β (pg/ml)**				
preoperative sample [median (Q1–Q3)]	1.28 (0.72–1.68)	0.56 (0.31–1.00)	0.007	0.025
90 days follow-up sample [median (Q1–Q3)]	1.35 (0.54–1.71)	0.76 (0.40–1.64)	0.177	0.261
**IL-2 (pg/ml)**				
preoperative sample [median (Q1–Q3)]	1.13 (0.78–1.75)	0.78 (0.66–1.01)	0.010	0.023
90 days follow-up sample [median (Q1–Q3)]	1.48 (0.95–1.95)	1.01 (0.66–1.75)	0.186	0.644
**IL-3 (pg/ml)**				
preoperative sample [median (Q1–Q3)]	1.49 (0.90–2.38)	0.75 (0.36–1.35)	0.006	0.030
90 days follow-up sample [median (Q1–Q3)]	1.79 (0.80–2.25)	1.05 (0.48–1.47)	0.030	0.082
**IL-4 (pg/ml)**				
preoperative sample [median (Q1–Q3)]	6.27 (0.90–23.94)	0.9 (0.9–18.35)	0.505	0.699
90 days follow-up sample [median (Q1–Q3)]	11.83 (5.62–23.25)	14.21 (0.90–41.94)	0.808	0.397
IL-5 (pg/ml)				
preoperative sample [median (Q1–Q3)]	0.47 (0.08–1.06)	0.15 (0.05–0.32)	0.040	0.024
90 days follow-up sample [median (Q1–Q3)]	0.63 (0.12–1.11)	0.22 (0.05–0.52)	0.063	0.391
**IL-7 (pg/ml)**				
preoperative sample [median (Q1–Q3)]	2.70 (1.69–3.32)	0.78 (0.09–2.04)	0.002	0.029
90 days follow-up sample [median (Q1–Q3)]	3.22 (1.19–4.20)	1.45 (0.09–3.12)	0.030	0.162
**IL-8 (pg/ml)**				
preoperative sample [median (Q1–Q3)]	2.88 (2.13–4.06)	1.69 (1.13–2.76)	0.009	0.769
90 days follow-up sample [median (Q1–Q3)]	3.09 (1.82–4.53)	2.78 (1.35–3.55)	0.461	0.547
**IL-10 (pg/ml)**				
preoperative sample [median (Q1–Q3)]	0.38 (0.12–1.98)	0.12 (0.12–0.25)	0.037	0.111
90 days follow-up sample [median (Q1–Q3)]	0.25 (0.00–2.43)	0.00 (0.00–0.38)	0.082	0.799
**IL-15 (pg/ml)**				
preoperative sample [median (Q1–Q3)]	0.77 (0.24–2.22)	0.24 (0.02–0.77)	0.018	0.044
90 days follow-up sample [median (Q1–Q3)]	1.03 (0.35–2.47)	0.62 (0.35–1.55)	0.408	0.199
**MCP-1 (pg/ml)**				
preoperative sample [median (Q1–Q3)]	390.21 (250.25–490.61)	363.80 (284.93–470.04)	0.812	0.943
90 days follow-up sample [median (Q1–Q3)]	425.48 (328.68–580.35)	374.87 (312.11–424.63)	0.253	0.150
**MIP-1α (pg/ml)**				
preoperative sample [median (Q1–Q3)]	4.41 (2.67–5.48)	0.83 (0.48–4.70)	0.020	0.107
90 days follow-up sample [median (Q1–Q3)]	3.95 (0.83–5.63)	3.08 (0.83–4.96)	0.367	0.885
**MIP-1β (pg/ml)**				
preoperative sample [median (Q1–Q3)]	24.95 (11.94–32.76)	21.28 (15.51–26.09)	0.417	0.826
90 days follow-up sample [median (Q1–Q3)]	26.37 (3.81–33.36)	18.56 (15.51–27.62)	0.582	0.767
**TNF-α (pg/ml)**				
preoperative sample [median (Q1–Q3)]	15.73 (12.77–17.84)	15.52 (11.81–18.79)	0.944	0.332
90 days follow-up sample [median (Q1–Q3)]	15.75 (10.76–19.66)	14.62 (12.18–18.42)	0.849	0.365
**MIF (pg/ml)**				
preoperative sample [median (Q1–Q3)]	320.87 (238.10–452.86)	252.14 (195.26–452.89)	0.165	0.332
90 days follow-up sample [median (Q1–Q3)]	381.03 (245.09–751.89)	320.87 (245.09–491.43)	0.285	0.936
**tPAI (ng/ml)**				
preoperative sample [median (Q1–Q3)]	73.74 (46.99–113.98)	68.67 (57.23–85.23)	0.422	0.252
90 days follow-up sample [median (Q1–Q3)]	77.72 (49.36–97.36)	58.61 (37.94–81.31)	0.157	0.149
**sICAM-1 (ng/ml)**				
preoperative sample [median (Q1–Q3)]	180.88 (151.20–222.83)	155.02 (136.44–205.24)	0.285	0.806
90 days follow-up sample [median (Q1–Q3)]	184.08 (138.59–269.17)	170.56 (157.21–201.75)	0.666	0.769
**sVCAM-1 (ng/ml)**				
preoperative sample [median (Q1–Q3)]	562.26 (414.00–703.18)	462.06 (377.61–579.36)	0.144	0.381
90 days follow-up sample [median (Q1–Q3)]	571.25 (470.25–600.32)	565.78 (450.10–677.52)	0.542	0.380

P-values were calculated using Student’s *t* test or Mann–Whitney *U* test as appropriate and binary logistic regression analysis adjusted for grade of stenosis, *ESRS* antithrombotic pre-treatment and statin pre-treatment, *aCS* asymptomatic carotid artery stenosis, *Q1–Q3* quartile 1–quartile 3, *sCS* symptomatic carotid artery stenosis.

All cytokines with significant differences in binary logistic regression analysis showed a positive correlation to concentrations in the follow-up sample at 90 days [IL-2 (p < 0.001, r = 0.530), IFN-α2 (p < 0.001, r = 0.565), Fractalkine/CX_3_CL1 (p = 0.009, r = 0.408), IL-1β (p = 0.002, r = 0.467), IL-3 (p = 0.001, r = 0.499), IL-7 (p = 0.022, r = 0.362)]. See supplemental table S2A and B for inter-cytokine correlations in preoperative samples.

### Biomarker levels at 90 days follow-up

**Figure 3:**
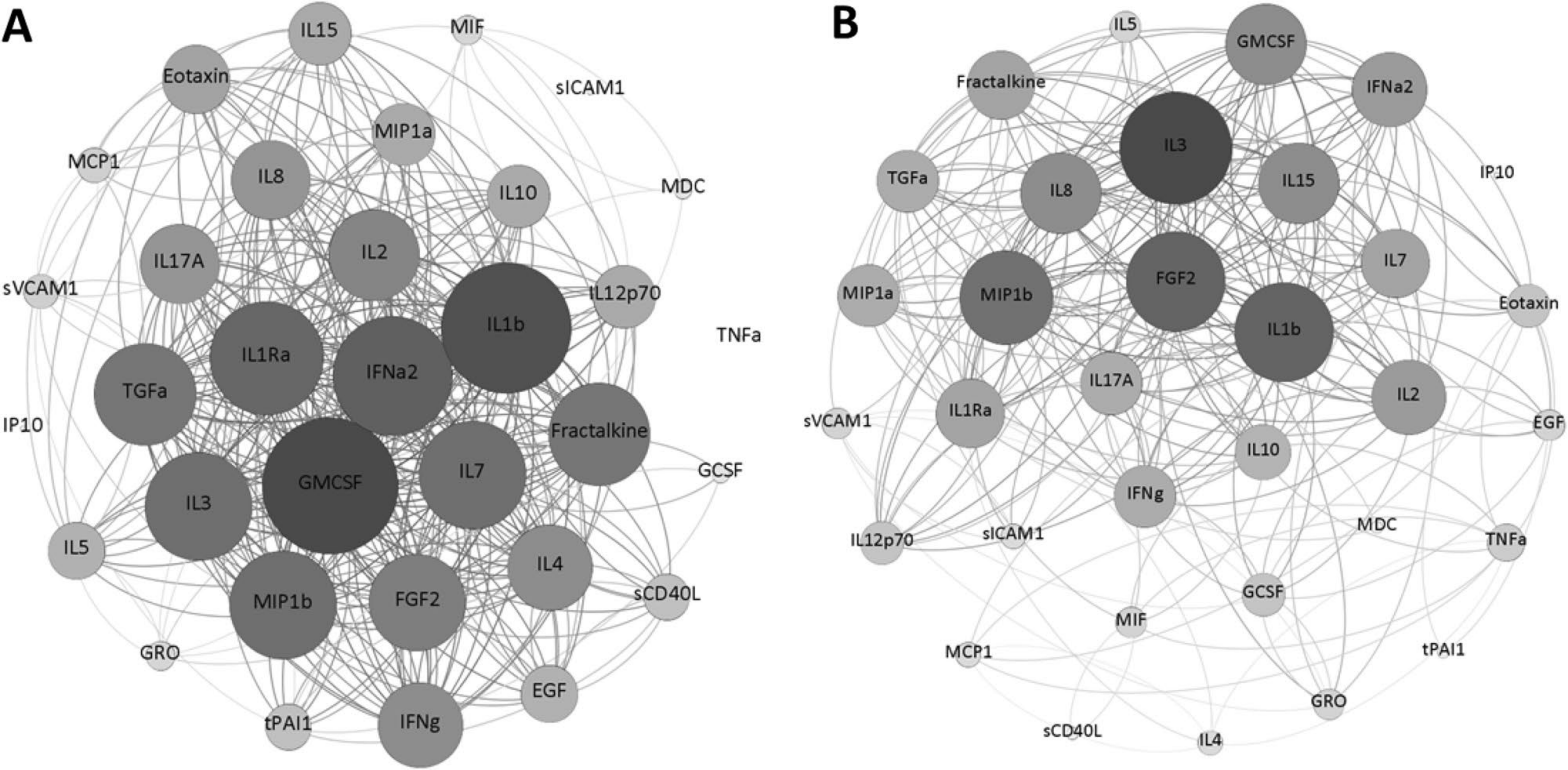
Inter-cytokine correlation networks in symptomatic and asymptomatic carotid stenosis. Force-directed graphs using Fruchtermann–Reingold-Algorithm depicting plasma inter-cytokine correlations in symptomatic (**A**) and asymptomatic (**B**) carotid stenosis at 90 days follow-up.

The univariable analysis revealed significantly higher plasma levels in patients with sCS at 90 days for the following markers: EGF (p = 0.010), IFN-a2 (p = 0.043), IL-3 (p = 0.030), IL-7 (p = 0.030). A trend towards higher levels could be detected for Fractalkine (p = 0.071), TGF-α (p = 0.075), G-CSF (p = 0.080), GM-CSF (p = 0.090), IL-5 (p = 0.063), IL-10 (p = 0.082) and sCD40L (p = 0.091). These group differences did not sustain to be significantly different after Benjamini–Hochberg-procedure and binary logistic regression analysis. However, there were distinct inter-cytokine correlation patterns in both groups which are visualised using Force-directed graphs in Fig. 3. For detailed information see the heat map of inter-cytokine correlations in the supplemental tables S2C and D.

### Histological analysis

The histological sum score was associated with presence of arterial hypertension (p = 0.013) and dyslipoproteinemia (p = 0.013) but did not show a group difference between sCS and aCS (p = 0.985). There were no significant differences between sCS and aCS regarding the investigated histological features. For detailed results of the histological analysis see supplemental table S1. Moreover, no differences regarding circulating biomarker concentrations were found between patients with plaques exhibiting more or less vulnerability criteria.

**Figure 4:**
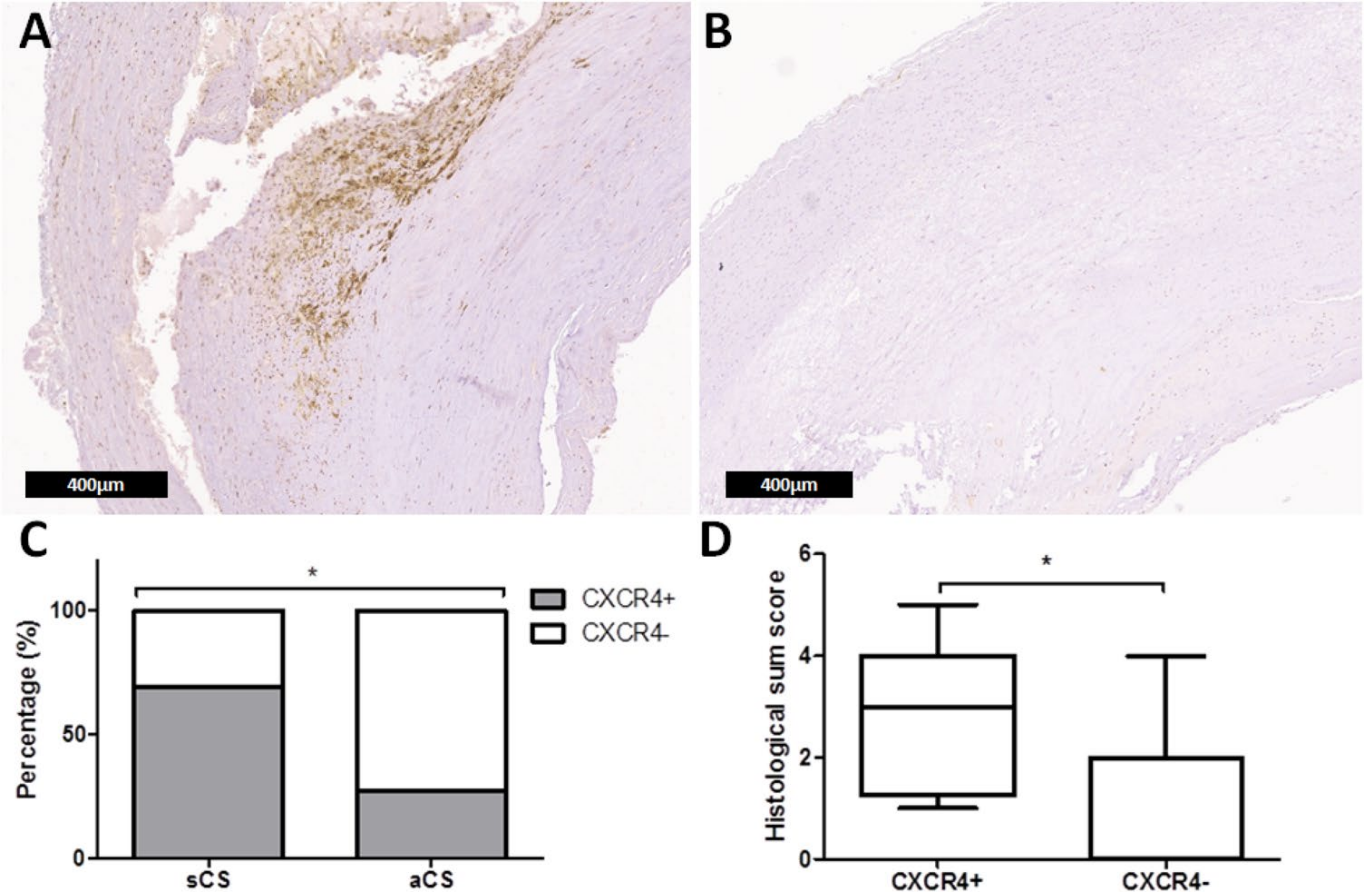
CXCR4-staining and relation to groups and criteria of plaque vulnerability. Exemplary CXCR4-staining of carotid plaque specimens of a sCS patient (scale bar: 400 µm) (**A**) and an aCS patient (scale bar: 400 µm) (**B**) indicating presence of CXCR4-positive inflammatory cell infiltrations in (**A**). Differential presence of CXCR4-positive inflammatory cells regarding groups (**C**) and histological sum score (**D**).

Immunohistochemistry revealed a significant difference for CXCR4-positive inflammatory cells in sCS compared to aCS (p = 0.034) (Fig. 4A–C). CXCR4-positivity on inflammatory cells was moreover associated with a higher histological sum score (p = 0.018) (Fig. 4D). In particular, CXCR4-positive inflammatory cells were related to a larger lipid core (p = 0.027) and chronic inflammation (p = 0.006). An association by trend existed for intraplaque hemorrhage (p = 0.053) which was more often present in plaques with CXCR4-positive inflammatory cells. There was no association between CXCR4-positivity and plasma cytokine levels nor an association between cytokine plasma concentrations and the histological sum score.

## Discussion

Cytokine mediated inflammatory mechanisms are crucial in atherosclerotic plaque vulnerability as well as in atherothrombosis. Targeting inflammatory biomarkers is a promising strategy in preventive treatment of vascular disease^11^. Indeed, the inhibition of IL-1β by Canakinumab investigated in the Canakinumab Antiinflammatory Thrombosis Outcome Study (CANTOS) trial led to a significant reduction of vascular events in patients with previous myocardial infarction^12^. Detailed knowledge of the mediators involved in the occurrence of cerebrovascular events is therefore of high interest. Previous studies focused on inflammatory markers within the atherosclerotic plaque and revealed distinct cytokine signatures associated with plaque vulnerability^13^. Likewise, numerous studies investigated circulating biomarkers regarding presence of carotid stenosis or measures of cerebrovascular risk^14^. The question remains whether systemic inflammatory markers appropriately reflect local plaque inflammation^15,16^ or rather an atherothrombotic event leading to cerebrovascular symptoms. To answer this question, we chose a comprehensive approach in order to identify a systemic cytokine pattern that is associated with characteristics of vulnerability or with the occurrence of ischaemic cerebrovascular events in carotid artery stenosis.

The analysis of patients’ baseline characteristics revealed significant differences regarding cardiovascular risk factors as well as antithrombotic and statin pretreatment, which were more common in patients with aCS. Due to preexisting cardiovascular diseases such as former coronary artery disease or myocardial infarction, these patients probably underwent more intensive prophylactic diagnostics in terms of check-up examinations, which lead to the diagnosis of aCS at an early and so far asymptomatic state. Compared to sCS patients with so far undiagnosed carotid artery stenosis and thus missing medical pretreatment, the asymptomatic group was also more likely to receive optimised medical treatment i.e. antithrombotic as well as lipid lowering therapy at baseline. This underscores the importance of risk stratification in terms of primary prevention. In sCS patients, moreover, male sex tended to be more prevalent than in aCS patients. This is in line with previous reports showing that carotid plaques of female patients might feature stable and less inflammatory plaque characteristics more frequently than those of male patients^17^.

Unexpectedly, we found no significant difference between sCS and aCS regarding the count of MES. Although there was a wider range of MES/h with a maximum count of 13 MES/h in sCS this difference did not reach statistical significance which may be due to the low rate of microembolisation and again the intensified medical treatment^18^ which has been in most cases initiated preceding Doppler sonography. Remarkably, especially statin treatment was previously reported being associated with lower counts of MES^19^.

The histological analysis revealed no significant group differences concerning established features of plaque vulnerability. Importantly, it must be kept in mind that all patients, i.e. also the aCS patients, suffered from advanced atherosclerotic disease and thus could feature a vulnerable plaque, even if this was so far not symptomatic. However, in the immunohistochemical staining, we detected a group difference regarding presence of CXCR4-positive inflammatory cells which were more prevalent in sCS plaques. This is clearly in line with a previous work by our group in which we investigated carotid plaque specimens by autoradiography using the ^68^Ga-Pentixafor tracer which specifically binds to CXCR4^20^. Consistently, we now could prove that CXCR4-presence is associated with known characteristics of plaque vulnerability. Merckelbach et al. reported a higher CXCR4-expression in advanced atherosclerotic plaques and that CXCR4 is predominantly expressed in macrophages^21^. While its specific function in atherosclerosis remains controversial^22^ targeting CXCR4 using molecular imaging might thus constitute a promising diagnostic strategy. In line with this, clinical positron emission tomography using ^68^Ga-Pentixafor was already evaluated in atherosclerotic diseases for the purpose of plaque imaging^20,23^.

The main finding of this study is the identification of a distinct cytokine pattern with significantly elevated plasma levels in sCS compared to aCS patients. After correction for multiple comparisons and adjustment for degree of stenosis, ESRS, antithrombotic pretreatment and statin pretreatment significant differences were observed for Fractalkine, IFN-α2, IL-1β, IL-2, IL-3 and IL-7. It is important to note that in this study we have placed particular emphasis on avoiding influence by stroke-induced inflammation. In the sCS group the median NIHSS was 0, seven patients had transient symptoms, and four patients suffered central retinal artery occlusion. Thus, influence on cytokine levels due to an inflammatory response by cerebral infarction is very unlikely. All of the mediators found to be significantly elevated have previously been implicated in cardiovascular disorders and were to some extent already tested in pre-clinical and clinical studies using antagonising antibodies.

The most prominent representative is probably IL-1β for which there is broad evidence regarding its role in atherosclerosis (for a review see^24^). As described above, IL-1β-antagonisation led to a significant reduction of vascular events in atherosclerotic diseased patients in the CANTOS-trial^12^. Additionally, IL-1β is suspected to support prothrombotic states^25^ and leads to platelet activation. Those platelets in turn secrete IL-1β^26^ which plays a fundamental role in adhesion to endothelial cells^27^. Thus, our result of an independent elevation of IL-1β in patients with sCS is clearly in line with the current evidence. However, clinical data on targeting IL-1β for the purpose of secondary stroke prevention are lacking so far.

Elkind et al. reported an independent association of IL-2 levels with carotid intima-media-thickness^28^. Others showed that treatment using an IL-2/anti-IL-2 monoclonal antibody (mAb) attenuates atherosclerosis via expansion of regulatory T cells (Tregs)^29^ and evidence exists that also Type 2 innate lymphoid cells might play an important role in IL-2 mediated reduction of atherosclerosis^30^. In summary it is postulated that IL-2 leads to secretion of IL-10 and TGF-β by regulatory T cells and independent secretion of IL-5 and IL13 by innate lymphoid cells, resulting in an overall reduction of plaque inflammation^30^. Thus, it will be interesting to see if immunomodulation via IL-2 might emerge as a potential therapy to limit atherosclerotic acceleration^31^.

IL-7 is a crucial mediator of T cell development and is contributing to endothelial inflammation via inducing monocyte recruitment^32^. Previously, Damås et al. showed an elevation of IL-7 levels in patients with unstable angina pectoris compared to patients with stable angina pectoris and controls^33^. Besides colony stimulating factors like G-CSF or GM-CSF, IL-3 is thought to stimulate haematopoiesis, especially in emergent settings (i.e. the differentiation of myeloid cells upon infection or inflammation)^34^. Robbins et al. reported that IL-3 and GM-CSF promote extramedullary differentiation of haematopoietic stem and progenitor cells to inflammatory monocytes which infiltrate and aggravate atherosclerotic lesions^35^. Of note, in our correlation analysis IL-3 was strongly associated with GM-CSF levels in both sCS and aCS patients. Unsurprisingly, GM-CSF did exhibit higher values in the sCS group. However, after Benjamini–Hochberg-correction this difference was not considered statistically significant. Because of its distinct functions in haematopoiesis and constitutive effects of immune cell influx in atherosclerosis, IL-3 might represent another therapeutic target^36^. Our results now indicate for the first time that these properties might be relevant for cerebrovascular events in patients with carotid stenosis.

Much less evidence about its inert biological function is available for IFN-α2 and our study is the first implicating this cytokine in carotid artery disease. However, IFN-α2 levels have previously been investigated in auto-immunological, haematological diseases and viral infections^37^. In cancer therapy, IFN-α2 leads to pericyte mediated vessel maturation and consequent stabilisation^38^. As we have previously shown that pericytes promote vascular stability in a model of atherosclerotic disease^39^, it will be interesting to investigate this interrelation in the future, in particular as IFN-based therapy was independently associated with reduced stroke risk in Hepatitis C patients^40^.

Fractalkine is a unique representative of chemokines in several respects. It exists in a soluble and membrane-bound form and fulfills chemotactic and adhesive functions for several inflammatory cell populations, especially monocytes^41^. In previous studies Fractalkine was implicated in atheroma growth and suspected to contribute to formation of platelet-monocyte aggregates suggesting a detrimental role in atherosclerotic diseases^41,42^. By contrast, Getzin et al. showed that Fractalkine and its receptor, CX_3_CR1, are involved in regeneration of the endothelium after arterial injury via recruitment of non-classical monocytes^43^. In concordance to these rather beneficial properties experimental studies identified Fractalkine as being neuroprotective in different brain disorders^44,45^, including stroke^46^. Likewise, we and others described an inverse association of Fractalkine/CX_3_CL1 dynamics with clinical severity and outcome in stroke patients^47,48^. Thus, it remains elusive whether the significant elevation of Fractalkine levels in this study is to be interpreted as primarily hazardous or as compensatory regenerative mechanism. In a previous study, we investigated monocyte subsets and related chemokines, i.e. Fractalkine/CX_3_CL1 and MCP-1/CCL2, in patients with sCS, aCS or cardioembolic stroke^49^. In contrast to our current findings, in the previous study we observed different levels of MCP-1/CCL2, but not of Fractalkine/CX_3_CL1. These divergent results could originate from the inclusion of patients with higher stroke severity in the previous study, whereas we abstained from this as far as possible in the current study for the reasons stated above. Of note, MCP-1/CCL2 was recently identified as biomarker of stroke risk in a large meta-analysis^50^. The role of MCP-1/CCL2 in atherosclerosis is meanwhile clearly established^51^. Thus, one might speculate that MCP-1/CCL2 increase might be less attributable to atherothrombosis than to cerebral tissue damage, as also murine stroke studies suggest^52^.

We did not find any association of circulating cytokines with histological features of plaque vulnerability or with MES. After correction for multiple comparisons no significant group differences in plasma biomarker levels could be observed at the three months follow-up. Thus, it seems very likely that the biomarker changes presented are attributable to the atherothrombotic event rather than to plaque vulnerability. However, the inter-cytokine correlation analysis revealed a distinct network of markers that was remarkably divergent between groups also at follow-up. This potentially indicates a persisting systemic pro-inflammatory milieu in sCS patients which in turn may contribute to an increased vascular risk^5^. In order to further investigate this hypothesis, we propose longitudinal biomarker studies in patients with significant carotid atherosclerosis to evaluate the prognostic value of the presented cytokines. If the hypothesis that the network patterns presented might constitute a risk factor for atherothrombotic events in carotid stenosis, this would be of interest not only for diagnostic, but also for therapeutic purposes.

This study has several limitations. We comprehensively characterised patients resulting in a large dataset in a relatively small sample size. Although, we applied corrections for multiple comparisons and logistic regression analysis, according studies with larger sample sizes are clearly warranted to validate our findings. Since this is an observational study, we are not able to conclude any causal relation of the biomarkers with clinical endpoints but only associations.

## Conclusions

In conclusion, in our analysis adjusted for vascular risk factors, grade of stenosis, antithrombotic and statin pretreatment we identified distinct cytokines being elevated in patients who suffered cerebrovascular events due to carotid stenosis. Of note, these mediators exhibit various functions concerning atherosclerotic inflammation, platelet activation or inflammatory cell recruitment and haematopoiesis. While we did not find an association of these circulating biomarkers with features of plaque vulnerability, presence of CXCR4-positive inflammatory cells was associated both with sCS and vulnerability characteristics. However, systemic inter-cytokine correlations were distinct and pronounced in sCS compared with aCS patients even at a three months follow-up, probably indicating a persisting pro-inflammatory milieu. Further, larger and longitudinal studies are warranted in order to clarify the diagnostic and therapeutic value of the mediators presented.

## Supplementary Information

Supplementary Information.

## Data Availability

The datasets generated during and analysed during the current study are available from the corresponding author on reasonable request.
