# Supplementary material for: Distinct systemic cytokine networks in symptomatic and asymptomatic carotid stenosis
Source: Sci Rep. 2020 Dec 15;10:21963. doi: 10.1038/s41598-020-78941-8 (PMC7738491; doi:10.1038/s41598-020-78941-8)
Supplement: Supplementary file 1 — Supplementary Information. [file 41598_2020_78941_MOESM1_ESM.docx]

**Online Supplement**

**Distinct Systemic Cytokine Networks in Symptomatic and Asymptomatic Carotid Stenosis**

Ricarda D. Stauss^A*^, Gerrit M. Grosse^A*^, Lavinia Neubert^B^, Christine S. Falk^C^, Danny Jonigk^B^, Mark P. Kühnel^B^, Maria M. Gabriel^A^, Ramona Schuppner^A^, Ralf Lichtinghagen^D^, Mathias Wilhelmi^E,F^, Karin Weissenborn^A*^, Claudia Schrimpf^F,G*^

^A^Department of Neurology, Hannover Medical School, Hannover, Germany

^B^Institute of Pathology, Hannover Medical School, Hannover, Germany

^C^Institute of Transplant Immunology, Hannover Medical School, Hannover, Germany
^D^Institute of Clinical Chemistry, Hannover Medical School, Hannover, Germany
^E^Department of Vascular- and Endovascular Surgery, St. Bernward Hospital, Hildesheim, Germany
^F^Division of Vascular and Endovascular Surgery, Department of Cardiothoracic-, Transplantation- and Vascular Surgery, Hannover Medical School, Hannover, Germany

^G^Department of Vascular Surgery, University Hospital Zurich, Zurich, Switzerland

*These authors contributed equally

**Address for correspondence**

Gerrit M. Grosse, MD

Department of Neurology

Hannover Medical School

Carl-Neuberg-Str. 1

30625 Hannover, Germany

Tel.: +49-511-532-3580; Fax: +49-511-532-18625

E-mail: grosse.gerrit@mh-hannover.de

ORCID iD: https://orcid.org/0000-0002-5335-9880

**Supplemental Table S1: Comparison of histological features in symptomatic and asymptomatic carotid plaques**

|  | **sCS** | | **aCS** | | p-value |  |
| --- | --- | --- | --- | --- | --- | --- |
|  | n= | % | n= | % |  |  |
| **Intima fibrosis** |  |  |  |  | p=0.626 | (sCS n=22, aCS n=24) |
| grade 1 | 0 |  | 1 |  |  |  |
| grade 2 | 1 |  | 1 |  |  |  |
| grade 3 | 21 |  | 22 |  |  |  |
| **Lipid core** |  |  |  |  | p=0.116 | (sCS n=22, aCS n=24) |
| grade 0 | 9 |  | 8 |  |  |  |
| grade 1 | 7 |  | 14 |  |  |  |
| grade 2 | 6 |  | 2 |  |  |  |
| **Calcification** |  |  |  |  | p=0.360 | (sCS n=22, aCS n=24) |
| grade 0 | 10 |  | 6 |  |  |  |
| grade 1 | 4 |  | 7 |  |  |  |
| grade 2 | 4 |  | 3 |  |  |  |
| grade 3 | 4 |  | 8 |  |  |  |
| **Media degeneration** |  |  |  |  | p=0.209 | (sCS n=17, aCS n=17) |
| grade 0 | 1 |  | 0 |  |  |  |
| grade 1 | 10 |  | 13 |  |  |  |
| grade 2 | 3 |  | 0 |  |  |  |
| grade 3 | 3 |  | 4 |  |  |  |
| **Chronic inflammation** |  |  |  |  | p=0.827 | (sCS n=22, aCS n=24) |
| grade 0 | 8 |  | 10 |  |  |  |
| grade 1 | 11 |  | 12 |  |  |  |
| grade 2 | 3 |  | 2 |  |  |  |
| **Cholesterol crystals** (dichotomized), % | 13 | (59.1) | 12 | (50.0) | p=0.536 | (sCS n=22, aCS n=24) |
| **Neovascularization** (dichotomized), % | 13 | (59.1) | 12 | (50.0) | p=0.536 | (sCS n=22, aCS n=24) |
| **Intraplaque hemorrhage** (dichotomized), % | 7 | (31.8) | 8 | (33.3) | p=0.913 | (sCS n=22, aCS n=24) |
| **Fibrous cap rupture** (dichotomized), % | 3 | (18.8) | 4 | (26.7) | p=0.598 | (sCS n=16, aCS n=15) |
| **Adherent thrombus** (dichotomized), % | 2 | (9.1) | 0 | (0) | p=0.131 | (sCS n=22, aCS n=24) |
| **CXCR4-positive macrophages**  (25th – 75th percentile) | 37.5 | (17.25-108) | 20.0 | (9-85) | p=0.175 | (sCS n=14, aCS n=15) |
| **CXCR4-positive inflammatory cells** (dichotomized), % | 11 | (78.6) | 5 | (38.5) | **p=0.034** | (sCS n=14, aCS n=13) |
| **Histological sum score ≥2** (dichotomized), % | 13 | (59.1) | 14 | (58.3) | p=0.985 | (sCS n=22, aCS n=24) |
| p<0.05 is considered significant, p-values were calculated using Chi-square test and Mann-Whitney U-test. The last column provides the number of specimens per group that could be considered for analyses. Of note: not all histological features were applicable in all samples, so that changes in sample size occur. | | | | | | |

**Supplemental Table S2: Inter-cytokine correlation heatmaps**

**(A)**
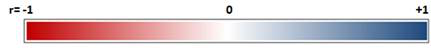


**(B)**
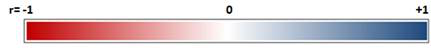


**(C)**
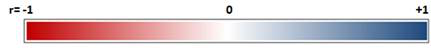


**(D)**
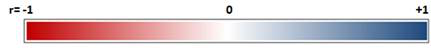


Heatmap of inter-cytokine correlations in (A) symptomatic carotid stenosis, preoperative blood sample, (B) asymptomatic carotid stenosis, preoperative blood sample, (C) symptomatic carotid stenosis at 90-day follow-up, (D) asymptomatic carotid stenosis at 90-day follow up. Correlation coefficients are shown as a color gradient from red (r=-1) to white (r=0) to blue (r=1). Significant correlations are bold printed. p<0.05 is considered significant. Analytes with less than 10 data points below minimum detection level have been excluded from correlation analysis.
